# A review of the genus *Berosus* Leach of Cuba (Coleoptera, Hydrophilidae)

**DOI:** 10.3897/zookeys.273.4591

**Published:** 2013-02-28

**Authors:** Albert Deler-Hernández, Martin Fikáček, Franklyn Cala-Riquelme

**Affiliations:** 1 Departamento de Zoología, Centro Oriental de Ecosistemas y Biodiversidad, Enramadas 601 esquina Barnada, Santiago de Cuba, 90100, Cuba; 2 Department of Entomology, National Museum, Kunratice 1, CZ-148 00 Praha 4, Czech Republic; 3 Department of Zoology, Faculty of Science, Charles University in Prague, Viničná 7, CZ-128 44 Praha 2, Czech Republic

**Keywords:** Hydrophilinae, Berosini, taxonomy, new synonymy, new records, Caribbean, Neotropical region, identification key

## Abstract

The Cuban fauna of the genus *Berosus* Leach, 1817 is reviewed based on newly collected material as well as historical and type specimens. Nine species are recognized, including three recorded from Cuba for the first time: *Berosus infuscatus* LeConte, 1855, *Berosus interstitialis* Knisch, 1924 (= *Berosus stribalus* Orchymont, 1946 **syn. n.**) and *Berosus metalliceps* Sharp, 1882. Only one of the nine Cuban species, *Berosus chevrolati*, remains endemic to Cuba, as two other species previously considered as endemic to Cuba are recorded from elsewhere: *Berosus quadridens* from Mexico and Central America and *Berosus trilobus* from the Dominican Republic. Notes on biology and Cuban distribution are provided for all nine species. *Berosus quadridens* Chevrolat, 1863, **stat. restit.** is removed from synonym with *Berosus truncatipennis* and considered a valid species.

## Abstract (es)

La fauna cubana de *Berosus* Leach, 1817 es revisada a partir de nuevo material colectado así, como de material tipo e histórico. Se reconocen un total de nueve especies, incluyendo tres nuevos registros: *Berosus infuscatus* LeConte, 1855, *Berosus interstitialis* Knisch, 1924 (= *Berosus stribalus* Orchymont, 1946, **syn. n.**) y *B metalliceps* Sharp, 1882. Solo una de las nueve especies cubanas, *Berosus chevrolati*, permanece como endémica, las otras dos especies originalmente consideradas como endémicas han sido registradas fuera de Cuba: *Berosus quadridens* presente en México y América Central y *Berosus trilobus* en República Dominicana. Se ofrecen datos sobre la biología y la distribución de todas las especies cubanas. *Berosus quadridens* Chevrolat, 1863, **stat. restit.** es destituida como sinónimo de *Berosus truncatipennis* y considerada como una especie válida.

## Introduction

The hydrophilid genus *Berosus* Leach, 1817 is the largest genus in family Hydrophilidae, containing more than 270 species distributed worldwide (Hansen 1999, Short and Fikáček 2011) and inhabiting various types of standing and slowly running waters (Oliva and Short 2012). The genus has been little studied in the Caribbean and in Cuba specifically, and the current knowledge is based primarily on occasional collecting events and historical records. Chevrolat (1863) described three species which are until now considered Cuban endemics: *Berosus trilobus* Chevrolat, 1863, *Berosus quadridens* Chevrolat, 1863 and *Berosus aculeatus* Chevrolat, 1863 (the name of the latter was later changed to *Berosus chevrolati* Zaitzev, 1908 due to the homonymy). Gundlach (1891) provided short redescriptions of these species and few additional records. Another supposedly endemic species, *Berosus stribalus* Orchymont, 1946, was described later by Orchymont (1946). Spangler (1973, 1981) recorded *Berosus undatus* (Fabricius, 1792) for the first time from Cuba and provided additional records on the five Cuban species. Hansen (1999) only listed four species of *Berosus* from Cuba. Finally, Peck (2005) published the most complete checklist of Cuban Coleopterawith data on their distribution; in this work he listed seven species of *Berosus*. Except of the published works, an unpublished thesis by Van Tassell (1966) contains additional data on Cuban *Berosus*, which we also adopt here.

In this paper we provide a review of the Cuban fauna of *Berosus* containing redescriptions of the three of four species described as Cuban endemics (*Berosus chevrolati*, *Berosus quadridens* and *Berosus trilobus*), we synonymize the fourth supposedly endemic species *Berosus stribalus* with a widely distributed Caribbean *Berosus interstitialis*, provide identification key and illustrations of all Cuban species and notes on their distribution and bionomics based on newly collected material. Three species are newly recorded for the Cuban fauna.

## Materials and methods

This study is mainly based on the material collected during the field survey of Cuban aquatic beetles conducted between 2008 to 2012 by A. Deler-Hernández, Y. S. Megna and F. Cala-Riquelme. The survey was mainly focused on eastern Cuba, but several areas of western Cuba were also sampled. In total, the samples from 170 localities have been collected, of which only 40 sites yielded *Berosus*. Specimens were collected with aquatic nets and preserved in 70%-95% ethanol. Except of this material, we also used the following sources of information: *i)* recently collected specimens provided to us by some Cuban colleagues; *ii)* material deposited in the zoological collection of the Instituto de Ecología y Sistemática in La Habana, Museo de Historia Natural “Charles T. Ramsden”, Universidad de Oriente in Santiago de Cuba, National Museum in Prague and the Division of Entomology of the University of Kansas in Lawrence; and *iii)* literature records (Chevrolat 1863; Gundlach 1891; Van Tassell 1966; Spangler 1973, 1981; Hansen 1999; Peck 2005). In the systematic section we provide detailed descriptions and differential diagnoses for three species originally described as Cuban endemics (*Berosus chevrolati*, *Berosus quadridens* and *Berosus trilobus*), for remaining species we only include a short diagnosis summarizing the most important diagnostic characters.

Habitus photographs were taken using Canon D-550 digital camera with attached Canon MP-E65mm f/2.8 1–5× macro lens, and subsequently adapted in Adobe Photoshop CS2. Photographs of genitalia were taken using Nikon Coolpix P6000 digital camera attached to Olympus BX41 compound microscope and subsequently combined with Helicon Focus software. Line drawings were traced from the photographs taken using a Canon PowerShot A620 camera attached to a Zeiss Stemi 2000-C stereomicroscope or with the same equipment as for taking the habitus photographs. Dissections of male genitalia and mounting techniques follow those used by Oliva and Short (2012). Complete label data are provided for type specimens, data of additional material are listed in an adapted form; our notes to the label data are in square brackets [ ]; and it is added the catalogue number for each vial of the Cuban material deposited in BSC-E. General morphological terminology follows Hansen (1991) and Komarek (2004), special terminology concerning *Berosus* follows Oliva (1989) and Oliva and Short (2012).

Examined material is deposited in the following collections:

BSC-E Departamento de Zoología, Centro Oriental de Ecosistemas y Biodiversidad, Santiago de Cuba, Cuba (A. Deler-Hernández);

CZACC Colección Zoológica, Instituto de Ecología y Sistemática, La Habana, Cuba (I. Fernández);

CZCTR Museo de Historia Natural “Charles Ramsden”, Facultad de Ciencias Naturales, Universidad de Oriente, Santiago de Cuba, Cuba (C. T. Ramsden historical collection) (M. Soto);

IRSNB Institut Royal des Sciences Naturelles de Belgique, Brussels, Belgium (P. Limbourg);

KSEM Division of Entomology, University of Kansas Natural History Museum, Lawrence, USA (A. Short);

NMPC National Museum, Prague, Czech Republic (M. Fikáček);

MNHN Museum National d’Histoire Naturelle, Paris, Frances (Bedel collection) (A. Mantilleri).

### Checklist of the Cuban species of *Berosus*

(asterisk indicates the species newly recorded for Cuba)

*Berosus chevrolati* Zaitzev, 1908

*Berosus exiguus* (Say, 1825)

*Berosus infuscatus LeConte, 1855

*Berosus interstitialis Knisch, 1924

= *Berosus stribalus* Orchymont, 1946, **syn. n.**

*Berosus metalliceps Sharp, 1882

*Berosus peregrinus* (Herbst, 1797)

*Berosus quadridens* Chevrolat, 1863 **stat. restit.**

*Berosus trilobus* Chevrolat, 1863

*Berosus undatus* (Fabricius, 1792)

## Systematics

### Genus Berosus

Leach, 1817

http://species-id.net/wiki/Berosus

#### Diagnosis.

Adults are mostly medium-sized, elongate, and strongly convex. Coloration of the body is brown to yellowish-brown, with or without dark spots on the pronotum and elytra. The head is strongly flexed down, eyes are protuberant, and antennae have 7 antennomeres. The elytral apex is entire or produced into one or two spines. The mesoventral process is usually laminar. The male protarsi are widened and have four tarsomeres; those of females have five tarsomeres. The middle and hind tibiae and tarsi bear a fringe of long natatory setae. Abdominal ventrite 5 has a rectangular (or less frequently semicircular) emargination posteriorly.

Among Cuban hydrophilid genera, *Berosus* may be easily identified by large globular eyes, scutellum longer that wide and middle and hind tibiae and tarsi with well developed fringe of long natatory setae.

### Species treatments

#### Berosus chevrolati

Zaitzev, 1908

http://species-id.net/wiki/Berosus_chevrolati

Figures 1a–g 11 12a

Berosus aculeatus Chevrolat, 1863: 207 (primary homonym of *Berosus aculeatus* LeConte, 1855). – Gundlach 1891: 48 (diagnosis and distribution).
Berosus (s.str.) *chevrolati* Zaitzev, 1908: 358 (replacement name for *Berosus aculeatus* Chevrolat, 1863). – Van Tassell 1966: 169 (unpublished PhD thesis: redescription, identification key). – Spangler 1981: 155 (diagnosis and distribution). – Hansen 1999: 84 (catalogue). – Peck 2005: 48 (checklist). – Epler 2010: 12.24 (notes on distribution).

##### Type locality.

Cuba.

##### Type material examined.

Holotype: female (MNHN): “aculeatus / Ch. Cuba // this must be / Chevr. unique type of aculeatus / PJS [= P. J. Spangler] 1966”.

##### Additional material examined.

**CUBA**: **Santiago de Cuba**: 3 exs. (dry-mounted) (NMPC): Dos Caminos, stream, 20°11'2.50"N, 75°46'17.7"W, 150 m a.s.l., 01.viii.2008, leg. A. Deler-Hernández., 1 ex. (dry-mounted) (BSC-E): El Vivero, 1.6 km E of Dos Caminos, 20°11'2.50"N, 75°46'17.7"W, 150 m a.s.l. Guaninicú river, 20–21.vi.2012, leg. Deler-Hernández & Fikáček; 1 ex. (in alcohol) (BSC-E): La Maya, Cuatro Caminos, remanso [backwater] 20°07'58"N, 75°34'01"W, 150 m a.s.l, 24.i.2008, leg. Y. S. Megna, 00019.

**Published Cuban records:** **Pinar del Río**: Quemado de Pineda (Spangler 1981). **Matanzas**: Este de Matanzas [Eastern Matanzas], Río Canimar (Gundlach, 1891). **Camagüey**: Río El Manantiales (Spangler 1981). **Sancti Spíritus**: Río Caburny (Spangler 1973); Arroyo Vega Grande (Spangler 1973). **Santiago de Cuba**: Contramaestre, Pozo Caliente, Río Contramaestre (Spangler 1981); II Frente, Arroyo Jarahueca (Spangler 1981); II Frente, Sabanilla, Río Mayarí (Spangler 1981); Río Ceiba (Spangler 1981); III Frente, Matías, Río Mogote (Spangler 1981). **Guantánamo**: La Tinta, Río Baracoa (Spangler 1981); Baracoa, Río Miel (Spangler 1973); Niceto Peréz, Arroyo de los Berros (Spangler 1981).

**Figure 1.**
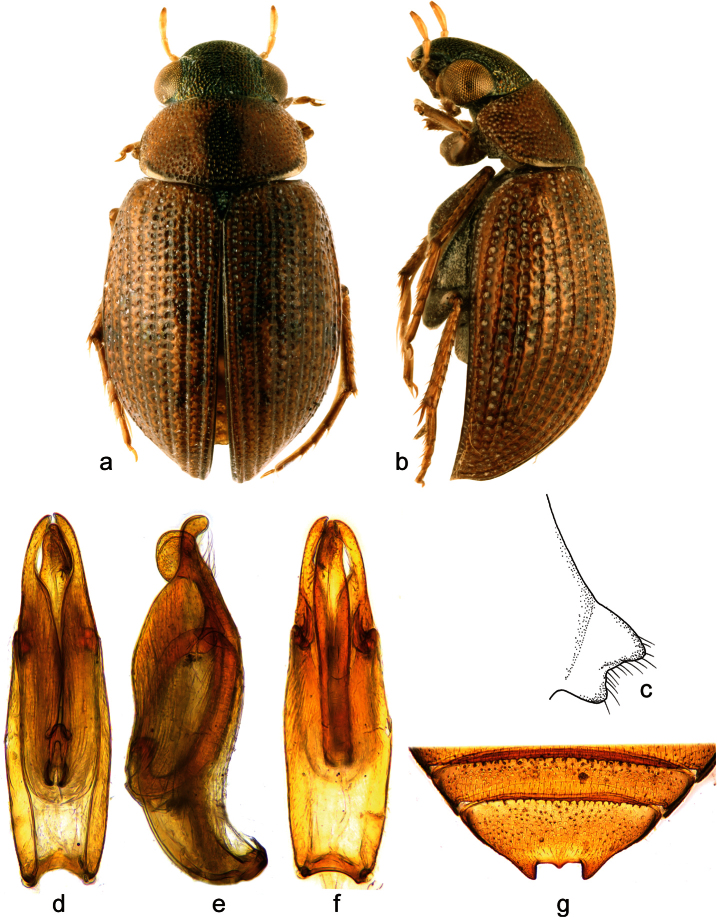
*Berosus chevrolati* Zaitzev, 1908. **a** habitus in dorsal view **b** habitus in lateral view **c** mesoventral process in lateral view **d-f** aedeagus (**d** dorsal view **e** lateral view **f** ventral view) **g** abdominal ventrite 5.

##### Diagnosis.

Small, widely elongate species, body length 3.6-4.6 mm. Head dark, metallic; pronotum pale, with median unpaired narrow black longitudinal spot mesally, pronotal punctation not darkened; elytra pale with irregular small dark spots in posterior half of elytral intervals. Elytral apices each without subapical tooth. Mesoventral process highly laminar, square-shaped, with large anterior and posterior teeth. Abdominal ventrite 1 with median keel throughout its length. Emargination of abdominal ventrite 5 rectangular with a median teeth. Median lobe of the aedeagus with short basal projection and rounded apex in lateral view.

##### Differential diagnosis.

*Berosus chevrolati* resembles *Berosus trilobus* (with which it may even co-occur) by the small strongly punctate body, metallic head, presence of an unpaired dark spot on the pronotum, mesoventrite with hooded anterior tooth, median keel developed throughout abdominal ventrite 1, emargination of abdominal ventrite 5 rectangular with single median tooth and the median lobe of the aedeagus with long basal lobe projecting far posteriad and enlarged apical portion in lateral view. It differs from *Berosus trilobus* by the narrow central dark spot on the pronotum (dark spot is large and trilobate in *Berosus trilobus*), elytra more evenly convex (subapical area of each elytra forms a bump in *Berosus trilobus*), shortbasal projection of the median lobe (long in *Berosus trilobus*) and, rounded apex of the median lobe in lateral view (apex is beak-shaped in lateral view in *Berosus trilobus*).

##### Redescription.

Habitus as in Figs 1a, b. Body length 3.4–4.6 mm. Body short and wide, moderately convex. Head black with metallic sheen, labrum black. Antennae testaceous. Maxillary palpi testaceous with palpomere 4 brown at apex. Pronotum testaceous with a central elongate metallic spot. Scutellum black with metallic sheen. Elytra testaceous with small brown spots without discrete borders. Pro-, meso- and metafemora testaceous, basal portion of metafemora sometimes slightly darker.

Head with moderately large and rounded punctures. Pronotum with punctures of the same size as on head. Scutellum with a few deeply impressed punctures slightly smaller than those on the pronotum. Elytral striae well-impressed. Interstriae with small and shallow punctures, irregular long setae on posterior half of elytra; spine-like setae absent. Elytral apices entire and rounded, of same shape in males and females. Mesoventral process highly raised, square-shaped, with hood-like anterior tooth, posterior tooth moderately large (Fig. 1c). Metaventral process wide, slightly raised, square-shaped, with large, deep glabrous rhomboid median depression; posterolateral angles raised and rounded, posteromesal projection carinate. Abdominal ventrite 1 with median carina throughout its length. Abdominal ventrite 5 with deep rectangular emargination, bearing a broad median tooth (Fig. 1g). Basal pubescence on basal 0.7 of meso- and of metafemora, the margin between pubescent and bare portions sinuate. Protarsus of male with adhesive soles on the first basal tarsomeres, first and second tarsomere distinctly thickened, third tarsomere very slightly thickened, fourth tarsomere elongate, almost as long as tarsomeres 1-3 combined. Claws moderately long, slender, arcuate.

Male genitalia (Figs 1d–f): Phallobase ca. 0.6× total length of aedeagus. Parameres in lateral view wide basally, apically projecting into rounded apex slightly bent ventrad, bearing a row of subapical setae ventrally. Median lobe C-shaped in lateral view; basal projection short, directing apicad; apex wide and rounded in lateral view.

##### Distribution.

Currently only known from Cuba. Spangler (1981) recorded this species from several localities across the island, but all new material is from two sites in Santiago de Cuba province.

##### Habitat.

We collected *Berosus chevrolati* along the margins of lowland streams and in isolated pools along these streams, in both cases having clear to turbid water and abundant organic matter (Fig. 11a). This species is found at low altitudes (ranging from sea level to ca. 160 m a.s.l.) situated in the Central Valley (Valle Central). *Berosus chevrolati* is frequently associated with *Berosus trilobus* in those habitats. Spangler (1981) also collected the species in standing waters.

#### Berosus exiguus

(Say, 1825)

http://species-id.net/wiki/Berosus_exiguus

Figures 2a–g 11

Hydrophilus exiguus Say, 1825: 189.
Berosus exiguus (Say). – Van Tassell 1966: 145 (unpublished PhD thesis: redescription, identification key, recorded from Cuba). – Testa and Lago 1994: 26 (diagnosis, bionomic and distribution notes, identification key). – Hansen 1999: 86 (catalogue). – Peck 2005: 48 (checklist). – Epler 2010: 12.19 (identification key, taxonomic notes). – Fernández et al. 2010: 28 (checklist). For complete references and synonymy see Hansen (1999)

##### Type locality.

USA: Virginia, Chincoteague Island.

##### Material examined.

**CUBA**: **Isla de la Juventud**: 1 ex. (in alcohol) (BSC-E): Punta del Este, Laguna Cayamás, 21°33'43"N, 82°33'18"W, 3 m a.s.l., 23.v.2006, leg. Y. S. Megna, 00165. **Camagüey**: 2 exs. (in alcohol) (BSC-E): Nuevitas, Cayo Sabinal, Laguna permanente [permanent pool], 21°38'6.1"N, 77°10'8.2"W, 5 m a.s.l., 06.v.2010, leg. O. Bello, 00153; 2 exs. (dry-mounted) (NMPC): Cayo Sabinal, permanent lagoon, 21°38'6.1"N, 77°10'8.2"W, 5 m a.s.l., 06.v.2010, leg. Y. Torres. **Santiago de Cuba:** 1 ex. (in alcohol) (BSC-E): San Miguel de Parada, Laguna temporal [temporal pool], 20°11'2.50"N, 75°46'17.7"W, 1 m a.s.l., 29.v.2009, leg. A. Deler-Hernández, 00136; 4 exs. (in alcohol) (BSC-E): San Miguel de Parada, Laguna temporal [temporal pool], 20°11'2.50"N, 75°46'17.7"W, 1 m a.s.l., 05.ix.2009, leg. A. Deler-Hernández, 00151; 2 exs. (dry-mounted) (NMPC): San Miguel de Parada, temporal lagoon, 20°11'2.50"N, 75°46'17.7"W, 1 m a.s.l, 05.ix.2009, leg. A. Deler-Hernández.

**Published Cuban records:** **Pinar del Rio:** unspecified locality (Van Tassell 1966: 149, Map 21).

**Figure 2.**
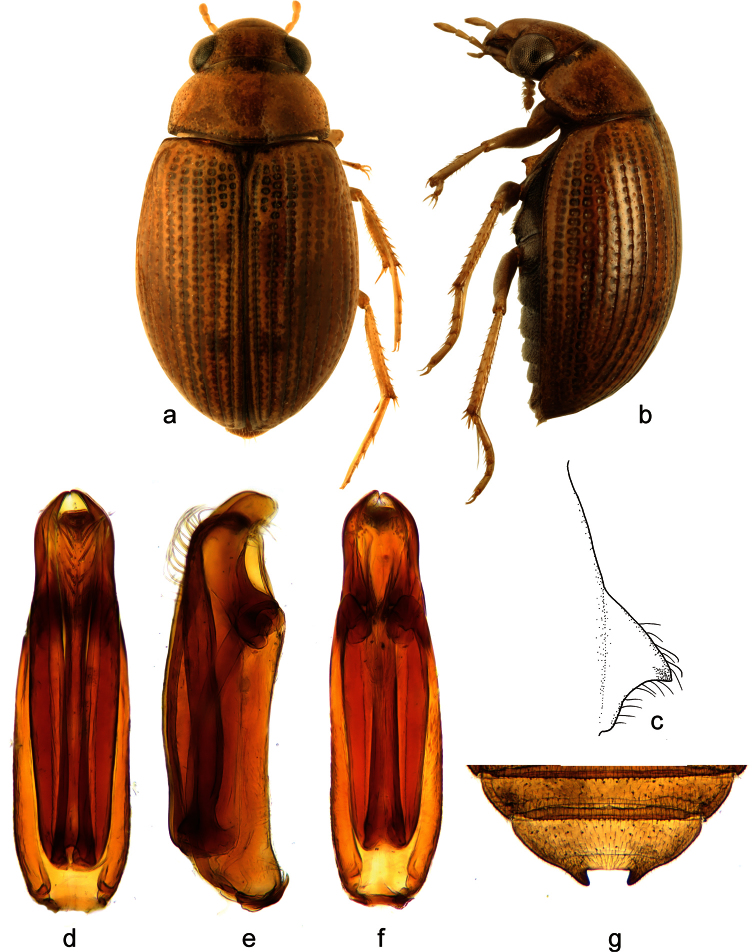
*Berosus exiguus* (Say, 1825). **a** habitus in dorsal view **b** habitus in lateral view **c** mesoventral process in lateral view **d–f** aedeagus (**d** dorsal view **e** lateral view **f** ventral view) **g** abdominal ventrite 5.

##### Diagnosis.

Habitus as in Figs 2a, b. Body length 3.0–3.7 mm. Head testaceuos, pronotum testaceous without median darker spots, punctation not darkened, elytra testaceous with irregularly arranged ill-defined slightly darker spots. Elytral apices entire and rounded in both sexes. Mesoventral process highly laminar, triangular in shape, anterior tooth large projecting posteriad (Fig. 2c). Abdominal ventrite 1 with median keel developed on basal half only. Emargination of abdominal ventrite 5 rectangular, without teeth (Fig. 2g) (in non-Cuban specimens, a very small medial tooth is present: Testa and Lago 1994). Aedeagus (Figs 2d–f) with median lobe only slightly shorter than parameres, with apex curved ventrad, bearing two series of long setae on dorsal surface.

##### Distribution.

Eastern USA (from New York to Florida, westwards reaching to Illinois, Indiana, Mississippi and Oklahoma), Bahamas (Young 1953; Hansen 1999; Peck 2005) and Cuba. In Cuba, it is known from the central and eastern region.

##### Habitat.

*Berosus exiguus* is mainly restricted to brackish waters in coastal regions. Cuban specimens have been collected in temporary brackish pools with clear water, abundant organic detritus on the bottom and associated aquatic riparian vegetation.

#### Berosus infuscatus

LeConte, 1855

http://species-id.net/wiki/Berosus_infuscatus

Figures 3a–g 11

Berosus infuscatus LeConte, 1855: 365: – Van Tassell 1966: 248 (unpublished PhD thesis: redescription, identification key). – Testa and Lago 1994: 26 (diagnosis, bionomic and distribution notes, identification key). – Epler 2010: 12.21 (identification key, taxonomic notes). For complete synonymy and references see Hansen (1999).

##### Type locality.

USA: “middle and southern States, e.g. New Orleans”.

##### Material examined.

**CUBA**: **Isla de la Juventud**: 1 ex. (in alcohol) (BSC-E): Punta del Este, Laguna temporal [temporal pool], 21°33'43"N, 82°33'18"W, 1 m a.s.l., 21.v.2006, leg. Y. S. Megna, 00160. **Pinar del Río**: 1 ex. (in alcohol) (BSC-E): Guanahacabibes, 21°54'26"N, 84°39'14"W, 3 m a.s.l., 20.iii.2003, leg. Y. S. Megna and O. Bello, 00176. **Camagüey**: 2 exs. (dry-mounted) (NMPC): Cayo Sabinal, lagoon, 21°38'6.1"N, 77°10'8.2"W, 5 m a.s.l., 06.v.2010, leg. Y. T. Cambas. **Las Tunas:** 1 ex. (in alcohol) (BSC-E): Palancón, 21°00'N, 76°54'W, 100 m a.s.l., 04.viii.2004, leg. Y. S. Megna, 00017. **Granma**: 1 ex. (in alcohol) (BSC-E): Cauto Cristo, Laguna permanente-I [permanent pool-I], 20°33'33.1"N, 76°28'44"W, 44 m a.s.l., 04.i.2005, leg. L. Chávez, 00175. **Guantánamo**: 1 ex. (dry-mounted) (CZACC): [no locality and date] leg. C. T. Ramsdem.

**Figure 3.**
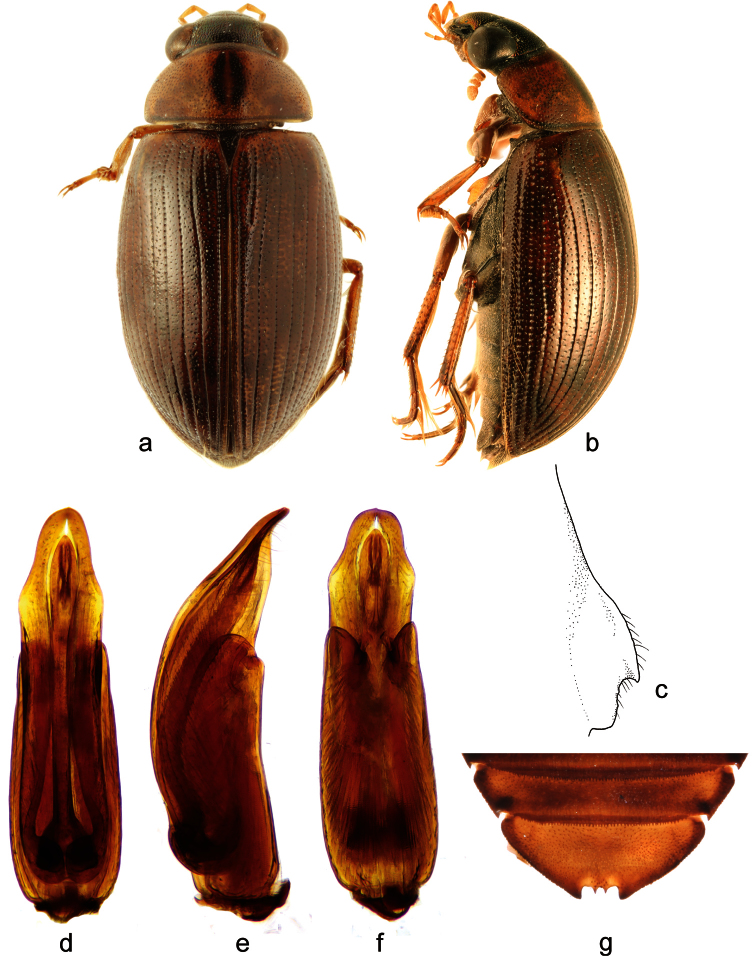
*Berosus infuscatus* LeConte, 1855. **a** habitus in dorsal view **b** habitus in lateral view **c** mesoventral process in lateral view **d–f** aedeagus (**d** dorsal view **e** lateral view **f** ventral view) **g** abdominal ventrite 5.

##### Diagnosis.

Habitus as in Figs 3a, b. Body length 5.5–6.0 mm. Head metallic black with paler anterior margin of clypeus; pronotum pale, with a pair of closely associated dark narrow longitudinal spots mesally, elytra brownish with indistinct irregularly arranged slightly darker spots. Head and pronotum with very distinct mesh-like microsculpture on interstices. Elytral apices entire and rounded in both sexes. Mesoventral process laminar, anterior tooth large, projecting posteriad (Fig. 3c). Abdominal ventrite 1 with median keel developed only between metacoxae. Emargination of abdominal ventrite 5 rectangular, with two sharp medial teeth (Fig. 3g). Aedeagus (Figs 3d–f) with median lobe slightly shorter than parameres, arched in lateral view. Parameres sinuate on lateral margin subapically.

##### Distribution.

USA (Alabama, Arkansas, Florida, Georgia, Illinois, Indiana, Louisiana, Mississippi, Missouri, North Carolina, Texas, Wisconsin), Mexico (Young 1953; Hansen 1999) and Cuba. The above specimens represent the first record of *Berosus infuscatus* from Cuba and the West Indies.

##### Habitat.

Cuban specimens of *Berosus infuscatus*have been collected among submerged aquatic vegetation in sun-exposed brackish permanent and temporary lagoons in coastal zones with turbid water and muddy/stony bottoms.

#### Berosus interstitialis

Knisch, 1924

http://species-id.net/wiki/Berosus_interstitialis

Figures 4a–g 11

Berosus tessellatus Fletiaux & Sallé, 1889: 337 (secondary homonym of *Enoplurus tessellatus* Motschulsky, 1859).
Berosus (s.str.) *interstitialis* Knisch, 1924: 270. – Van Tassell 1966: 191 (unpublished PhD thesis: redescription, identification key). – Epler 2010: 12.24 (taxonomic notes).
Berosus stribalus Orchymont, 1946: 13. **Syn. n.** – Spangler 1981: 156 (taxonomic and distribution notes). – Fernández et al. 2010: 28 (checklist).

##### Type locality.

Guadeloupe, Grande Terre and Trois-Rivières.

##### Type material examined.

*Berosus tessellatus*:Not examined. Type specimens were not found on our request for loan in MNHN. Instead, we examined the specimens identified as *Berosus tessellatus* and *Berosus interstitialis* deposited in coll. d’Orchymont and coll. Knisch in IRSNB (see below).

*Berosus stribalus*: Holotype: male (IRSNB): “[male sign] / Cuba 10. K / S. of Pinar Rio / Sep. 12-23 ’13 // A. d’Orchymont det. / Berosus (s.str.) / stribalus m. // Type”. Paratype: 1 spec. (IRSNB): “St. / Domin- / go // Berosus / striatus / Say // coll. Orch. // A. d’Orchymont det. / Berosus (s.str.) / stribalus m. // Para- / type”.

##### Additional material examined.

**CUBA: Isla de la Juventud**: 4 exs. (dry-mounted) (CZACC): vii.1960 [no locality and collector indicated]; 8 exs. (in alcohol) (BSC-E): Punta del Este, laguna temporal [temporal pool], 21°33'43"N, 82°33'18"W, 1 m a.s.l., 21.v.2006, leg. Y. S. Megna, 00180; 8 exs. (in alcohol) (BSC-E): Laguna Cayamás, 21°33'43"N, 82°33'18"W, 3 m a.s.l., 23.v.2006, leg. Y. S. Megna, 00178. **Pinar del Río**: 57 exs. (dry-mounted) (CZACC): Lomas de Soroa, v/vi.1963, [no collector indicated]; 1 ex. (in alcohol) (BSC-E): Viñales, arroyo [stream], 22°33'36.35"N, 83°49'59"W, 170 m a.s.l., 18.iv.2012, leg. A. Deler-Hernández, 00146. **Artemisa**:8 exs. (dry-mounted) (CZACC): Laguna Ariguanabo, vi.1963, [no collector indicated]. **Mayabeque**:1 ex. (dry-mounted) (CZACC): Jibacoa, littoral on north coast, v.1962, [no collector indicated]. **Matanzas**:1 ex. (dry-mounted) (CZACC): Playa Larga, iv.1965, [no collector indicated]; 1 ex. (dry-mounted) (CZACC): Bacunayagua, vi.1940 [no collector indicated]. **Camagüey**:1 ex. (in alcohol) (BSC-E): Sierra de Cubitas, Río El Roble, 21°32'53.23"N, 77°46'42.31"W, 55 m a.s.l., 14.iv.2012, leg. A. Deler-Hernández, 00148. **Las Tunas**:1 ex. (in alcohol) (BSC-E): Amancio, Comunales, laguna permanente [permanent pool], 20°49'59"N, 77°32'32"W, 34 m a.s.l., 04.x.2008, leg. Y. S. Megna, 00179; 1 ex. (in alcohol) (BSC-E): La Fé, laguna temporal [temporal pool], 20°49'17.7"N, 77°34'40.8"W, 50 m a.s.l., 18.xii.2008, leg. Y. S. Megna, 00147. **Granma**: 2 exs. (in alcohol) (BSC-E): Cauto Cristo, laguna permanente [permanent pool], 20°33'33.1"N, 76°28'44"W, 44 m a.s.l., 04.i.2005, leg. L. Chávez, 00150; 1 ex. (dry-mounted) (NMPC): Cauto Cristo, permanent lagoon 20°33'33.1"N, 76°28'44"W, 44 m a.s.l., 04.i.2005, leg. L. Chávez. **Santiago de Cuba**:1 ex. (in alcohol) (BSC-E): Guamá, La Mula, laguna permanente [permanent pool], 19°58'33.6"N, 76°46'4.8"W, 4 m a.s.l., 20.vi.2008, leg. A. Deler-Hernández, 00018. **Guantánamo**:1 ex. (dry-mounted) (CZCTR): Guantánamo, San Carlos [at light], 20°26'22"N, 74°42'31"W, 160 m a.s.l., 18.vii.1915, leg. C. T. Ramsden; 1 ex. (dry-mounted) (CZCTR): San Carlos [at light], 20°26'22"N, 74°42'31"W, 160 m a.s.l., 24.viii.1917, leg. C. T. Ramsden; 1 ex. (in alcohol) (BSC-E): San Antonio del Sur, Macambo, Río Macambo, 20°03'26.9"N, 74°44'15"W, 4 m a.s.l., 24.x.2008, leg. A. Deler-Hernández; 1 ex. (in alcohol) (BSC-E): Baracoa, Nibujón, laguna temporal [temporal pool], 20°30'8.6"N, 74°38'88"W, 8 m a.s.l., 03.ii.2010, leg. A. Deler-Hernández, 00149. 1 ex. (dry-mounted) (IRSNB): [without precise locality]:“Cuba / Gundlach // 1541 / 977 // Kniž det. / interstitialis”. **GUADELOUPE:** 1 ex. (dry-mounted) (IRSNB): “Guadeloupe / coll. A. d’Orchymont // Berosus s.str. / tessellatus / Fleut. & Salle // A. d’Orchymont det.” [based on attached note, d’Orchymont compared this specimen with one of the types of *Berosus tessellatus* from the collection of Fleutiaux which is currently lost and not available for reexamination; the specimen is a male, but has the abdomen destroyed by a dermestid larva]; 1 ex. (dry-mounted) (IRSNB): Trois Riviéres, leg. Dufau. **PUERTO RICO:** 1 ex. (dry-mounted) (IRSNB): [without detailed locality data], leg. Moritz. **VIRGIN ISLANDS:** 2 exs. (dry-mounted) (IRSNB): Saint Thomas, leg. C. Felsche.

**Published Cuban records:** **Pinar del Río**: Entronque de Manuel Sanguili (Spangler 1981). **Isla de la Juventud**: Laguna Base Julio Antonio Mella (Spangler 1981). **Holguín**: Gibara, Arroyo Landivar at Finca Pozón (Spangler 1981). **Santiago de Cuba**: Matías (Spangler 1981).

**Figure 4.**
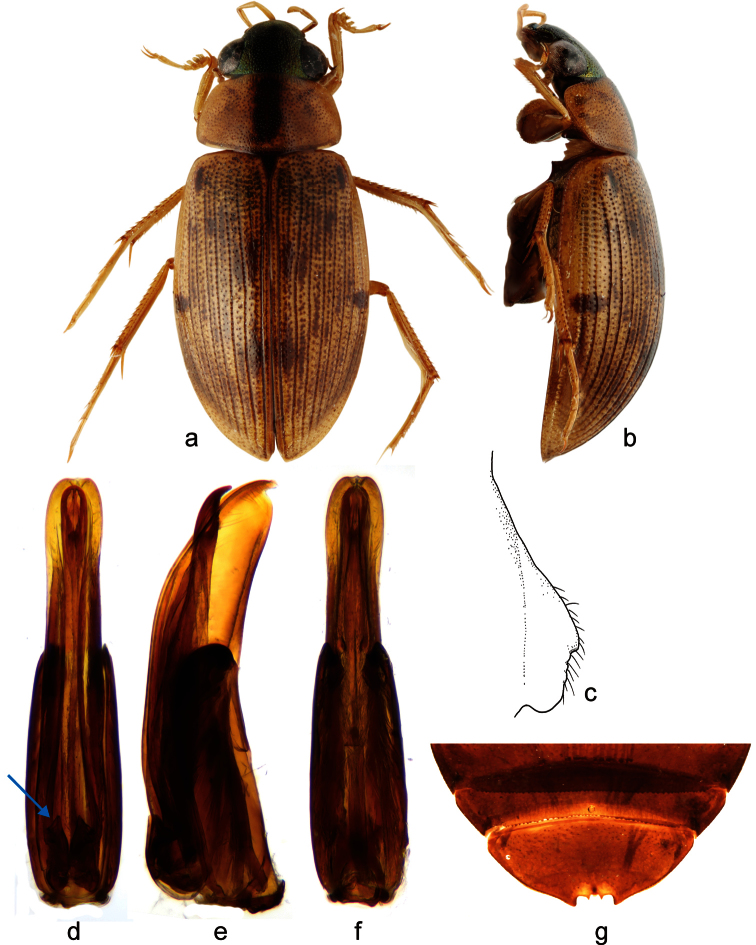
*Berosus interstitialis* Knisch, 1924. **a-b** habitus of the holotype of *Berosus stribalus* Orchymont, 1946 (**a** dorsal view **b** lateral view) **c** mesoventral process in lateral view **d–f** aedeagus (**d** dorsal view **e** lateral view **f** ventral view) **g** abdominal ventrite 5.

##### Diagnosis.

Habitus as in Figs 4a, b. Body length 5.0–5.3 mm. Head uniformly dark, metallic green; pronotum pale with a pair of closely aggregated longitudinal narrow dark spots mesally; elytra pale with darkened punctation and with dark spots in anterior and posterior third of intervals 1 and 2, in humeral area and at midlength of intervals 7-9, plus with variable number of spots on remaining intervals. Elytral apices entire in both sexes. Mesoventral process laminar, with small anterior tooth projecting ventrad, nearly straight middle portion and rounded posterior part (Fig. 4c). Abdominal ventrite 1 with median keel developed only between metacoxae. Emargination of ventrite 5 deep, subrectangular, with two slender medial teeth (Fig. 4g), not showing sexual dimorphism. Aedeagus (Figs 4d–f) strongly compressed from sides; parameres ca. 2× as long as phallobase, wide throughout in lateral view except for tooth-like apex; bases of the parameres in dorsal view with characteristic basal teeth.

##### Taxonomic note.

The synonymy of *Berosus stribalus* with *Berosus interstitialis* was first proposed in an unpublished thesis by Van Tassell (1966: 302). The reasons for the synonymy were not explained, and Cuba (i.e. type locality of *Berosus stribalus*) was not even mentioned in the distribution of *Berosus interstitialis* in the taxonomic part of the thesis. We were not able to examine the types of *Berosus tessellatus* from the collection of Fleutiaux in MNHN as the specimens were not found. We therefore examined the specimens identified as *Berosus tessellatus* and *Berosus interstitialis* deposited in IRSNB, including one male from Guadeloupe (type locality of *Berosus tessellatus*) bearing the note that it was compared with the types of *Berosus tessellatus* by A. d’Orchymont. Comparison of these specimens with the types of *Berosus stribalus* and with newly collected Cuban specimens revealed that they all specimens agree in the diagnostic characters mentioned above, including the characteristic shape of the aedeagus and a characteristic tooth on the base of each paramere. We may therefore confirm the unpublished synonymy proposed by Van Tassell (1966) and consider *Berosus stribalus* as a junior subjective synonym of *Berosus interstitialis*.

##### Habitat.

Cuban specimens were collected mainly in standing waters as well as in isolated pools along streams and rivers in the lowlands. The localities are usually exposed to sun and have turbid water, muddy bottom, submerged vegetation and are rich in organic matter.

##### Distribution.

Widely distributed Caribbean species, so far recorded from the Bahamas, Haiti Guadeloupe, Virgin Islands Puerto Rico and Cuba (Van Tassell 1966, Orchymont 1946, Epler 2010, this paper). The species is here recorded for the first time from Cuba, due to the synonymy of *Berosus stribalus* with *Berosus interstitialis*.

#### Berosus metalliceps

Sharp, 1882

http://species-id.net/wiki/Berosus_metalliceps

Figures 5a–g 11

Berosus metalliceps Sharp, 1882: 83. – Van Tassell 1966: 150 (unpublished PhD thesis: redescription, identification key). – Epler 2010: 12.24 (taxonomic and distribution notes). For complete synonymy and references see Hansen (1999).

##### Type locality.

México: Tres Marías Island.

##### Material examined.

**CUBA**: **Camagüey**:1 ex. (dry-mounted) (NMPC): Cayo Sabinal, permanent lagoon, 21°38'6.1"N, 77°10'8.2"W, 5 m a.s.l., 06.v.2010, leg. Y. Torres.

**Figure 5.**
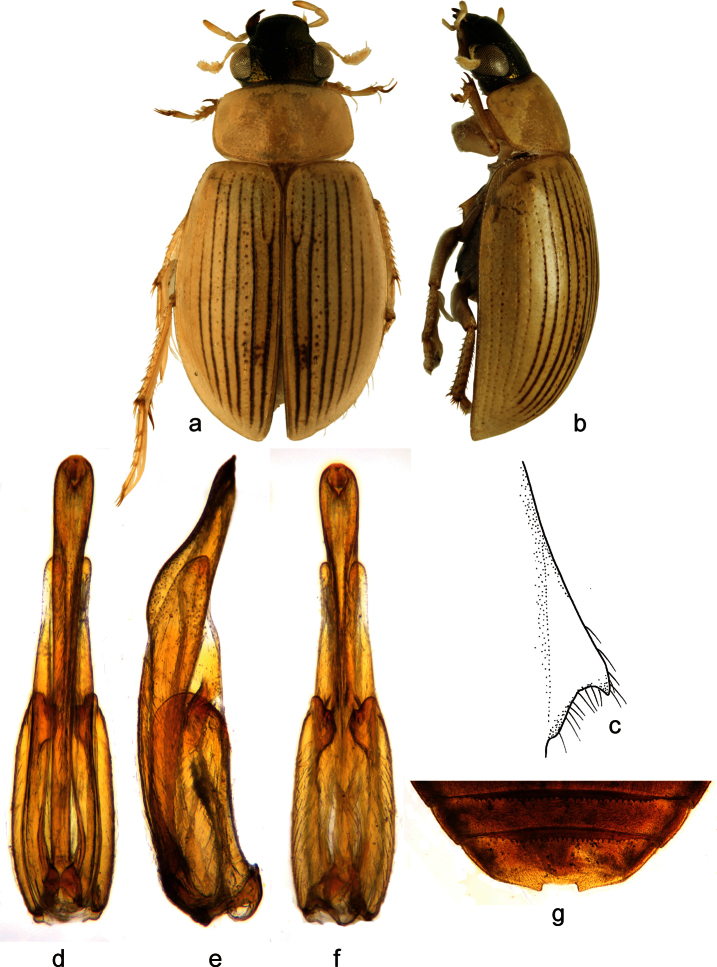
*Berosus metalliceps* Sharp, 1882. **a–b** habitus of the only known Cuban specimen (**a** dorsal view **b** lateral view) **c** mesoventral process in lateral view **d–f** aedeagus (**d** dorsal view **e** lateral view **f** ventral view) **g** abdominal ventrite 5.

##### Diagnosis.

Habitus as in Figs 5a, b. Body length 4.5 mm. Head metallic black, pronotum pale without dark spots, elytra pale with dark stripes on elytral series and slightly darker spot in posterior third of interval 1. Elytral apices entire and rounded. Mesoventral process laminar, triangular in shape, anterior tooth projecting posteriad (Fig. 5c). Abdominal ventrite 1 with median keel developed only between metacoxae. Emargination of abdominal ventrite 5 rectangular, without tooth median (Fig. 5g). Aedeagus (Figs 5d–f) with median lobe much longer than parameres, with enlarged spatula–shaped apex in ventral view, sinuate on dorsal face in lateral view. Parameres simple, rounded apically, phallobase ca. 0.3× total length of aedeagus.

##### Distribution.

USA (California), Mexico, Bahamas (Young 1953; Hansen 1999) and Cuba. The above specimen represents the first record of *Berosus metalliceps* from Cuba.

##### Habitat.

The Cuban specimen was collected in the highly exposed brackish permanent lagoon with muddy bottom.

#### Berosus peregrinus

(Herbst, 1797)

http://species-id.net/wiki/Berosus_peregrinus

Figures 6a–g

Hydrophilus peregrinus Herbst, 1797: 314.
Berosus peregrinus (Herbst); LeConte (1855: 364, transferred to Berosus). – Van Tassell 1966: 163 (unpublished PhD thesis: redescription, identification key). – Smetana 1988: 50 (diagnosis, recorded from Cuba). – Hansen 1999: 91 (catalogue). – Peck 2005: 48 (checklist). – Epler 2010: 12.20 (identification key).

##### Type locality.

“North America”.

##### Material examined.

**CUBA:** no material examined. **USA: Texas:** 1 ex. (dry-mounted) (NMPC); 1 ex. (in alcohol) (BSC-E): Maverick Co., Rt. 277 at Tequesquite Creek, large creek [AS-03-011], 31.viii.2003, leg. A. E. Z. Short.

**Published Cuban records:** **Cuba:** without specified locality (Smetana 1988). **Pinar del Río:** without specified locality (Peck 2005).

**Figure 6.**
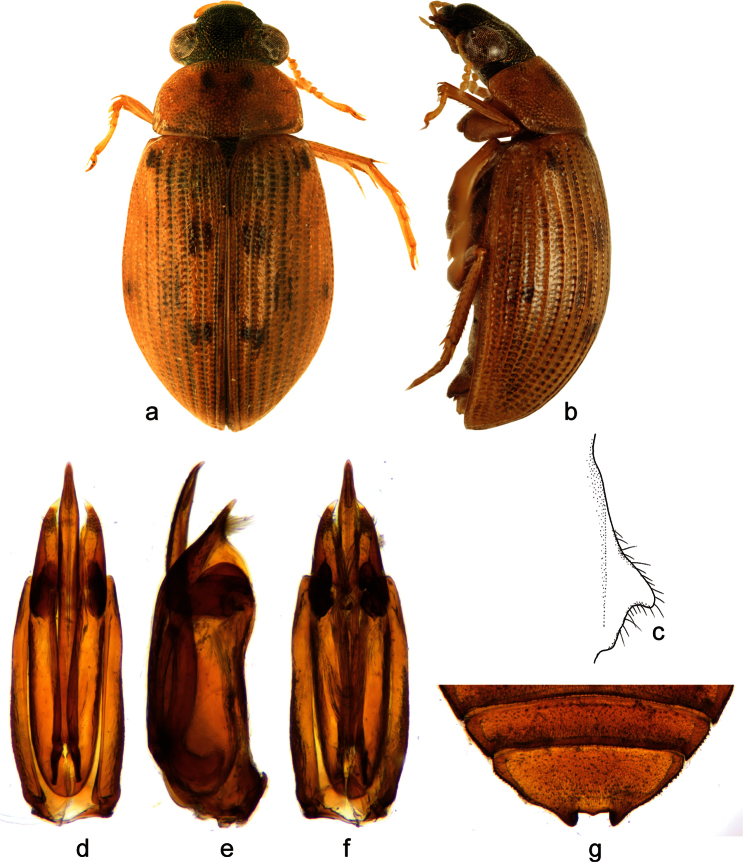
*Berosus peregrinus* (Herbst, 1797), specimen from USA, Texas. **a** habitus in dorsal view **b **habitus in lateral view **c** mesoventral process in lateral view **d–f** aedeagus (**d** dorsal view **e** lateral view **f** ventral view) **g** abdominal ventrite 5.

##### Diagnosis.

Habitus as in Figs 6a, b. Body length 4.1–5.2 mm. Head metallic black, pronotum pale with two small submedian dark spots anteriorly, elytra pale with rather sharply defined dark spots on intervals 1-2 and in humeral area. Elytral apices entire and rounded. Mesoventral process laminar, triangular in shape, anterior tooth large, projecting posteriad (Fig. 6c). Abdominal ventrite 1 with median keel developed only between metacoxae. Emargination of abdominal ventrite 5 rectangular with a single median broad and short tooth (Fig. 6g). Aedeagus (Figs 6d–f) with median lobe slender, pointed at apex, parameres shorter than median lobe, very wide in lateral view, narrowing into sharply pointed apex bearing tuft of setae apically. Phallobase long, ca. 0.6× total length of aedeagus.

##### Distribution.

Canada (Nova Scotia, Ontario, Quebec), USA (from New York and Pennsylvania to Florida, Louisiana, Mississippi and Texas, westward at least to Arizona, Illinois, Indiana and Wisconsin) (Hansen 1999), and Cuba. In Cuba, *Berosus peregrinus* has been recorded only from Pinar del Río (without exact locality) by Peck (2005). We did not collect this species in our survey.

#### Berosus quadridens

Chevrolat, 1863 stat. restit.

http://species-id.net/wiki/Berosus_quadridens

**Figure 7.**
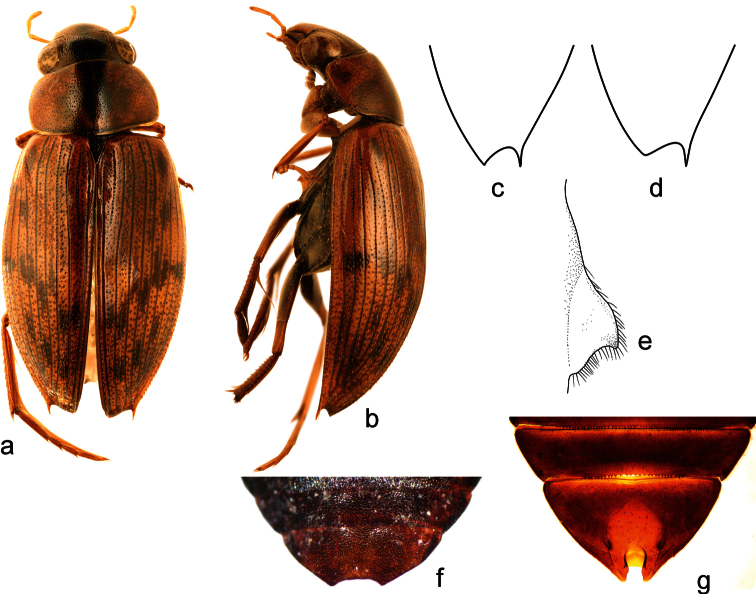
*Berosus quadridens* Chevrolat, 1863. **a** habitus in dorsal view **b** habitus in lateral view **c** apex offemale elytron **d** apex of male elytron **e** mesoventral process in lateral view **f–g** abdominal ventrite 5 (**f** female **g** male).

Figures 7a–g 8e–h 11

Berosus (Anchialus) quadridens Chevrolat, 1863: 206.
Berosus quadridens : – Gundlach 1891: 47 (diagnosis and distribution). – Zaitzev 1908: 357. – Mouchamps 1963: 121 (synonymized with *Berosus truncatipennis* Castelnau, 1840). – Van Tassell 1966: 56 (unpublished PhD thesis: redescription, identification key). – Spangler 1981: 156 (diagnosis and distribution). – Hansen 1999: 82 (as synonym of *Berosus truncatipennis*). – Peck 2005: 48 (checklist). – Deler-Hernández and Cala-Riquelme 2010: 73 (diagnosis, distribution, identification key).

##### Type locality.

Cuba.

##### Type material. 

Not examined.

**Additional material examined CUBA**: **Pinar del Río:** 1 ex. (dry-mounted) (IRSNB): S of Pinar del Rio, 12/23.ix.1913. **Isla de la Juventud**: 7 exs. (in alcohol) (BSC-E): Punta del Este, Laguna temporal [temporal pool], 21°33'43"N, 82°33'18"W, 1 m a.s.l., 21.v.2006, leg. Y. S. Megna, 00142. **Granma**: 3 exs. (in alcohol) (BSC-E): Cauto Cristo, Laguna permanente-I [permanent pool-I], 20°33'33.1"N, 76°28'44"W, 44 m a.s.l., 04.i.2005, leg. L. Chávez, 00087; 1 ex. (in alcohol) (BSC-E): Cauto Cristo, Laguna permanente-I [permanent pool-I], 20°33'33.1"N, 76°28'44"W, 44 m a.s.l., 13.vi.2004, leg. L. Chávez, 00174. **Santiago de Cuba**: 1 ex. (in alcohol) (BSC-E): Palma Soriano, Monte Barranca, 20°20'13.5"N, 76°1'11.6"W, 203 m a.s.l., 05.xii.2007, leg. A. Deler-Hernández and B. Téllez, 00052. **MEXICO**: **Sinaloa**:1 ex. (dry-mounted) (IRSNB): Los Mochis Station, x.1921 leg. R. V. van Zwaluwenburg. **Veracruz**:2 exs. (dry-mounted) (IRSNB): without more detailed locality, leg. Höge. **GUATEMALA:** 4 exs. (dry-mounted) (IRSNB): Paso Antonio, 400 ft., leg. Champion. **NICARAGUA: Chinandega:** 1 ex. (dry-mounted) (IRSNB): Posoltega, 06.v.1984, UV light, leg. Algodon. **COSTA RICA**: **Guanacaste**:10 exs. (dry-mounted) (KSEM, NMPC): 11.5 km W of Cañas, 15 m a.s.l., HG light by ditch/field [AS-04-026], leg. A. E. Z. Short & D. J. Lebbin; 1 ex. (dry-mounted) (NMPC): Highway 1, 13.1 km SW of Liberia, roadside ditch/pools, 16.vi.2003, leg. A. E. Z. Short.

**Published Cuban records:** **Cuba**: **Isla de la Juventud**: Laguna Base Julio Antonio Mella (Spangler 1981). **Matanzas: Cárdenas** (Gundlach 1891). **Holguín**: Gibara, Arroyo Landivar at Finca Pozón (Spangler 1981).

**Figure 8.**
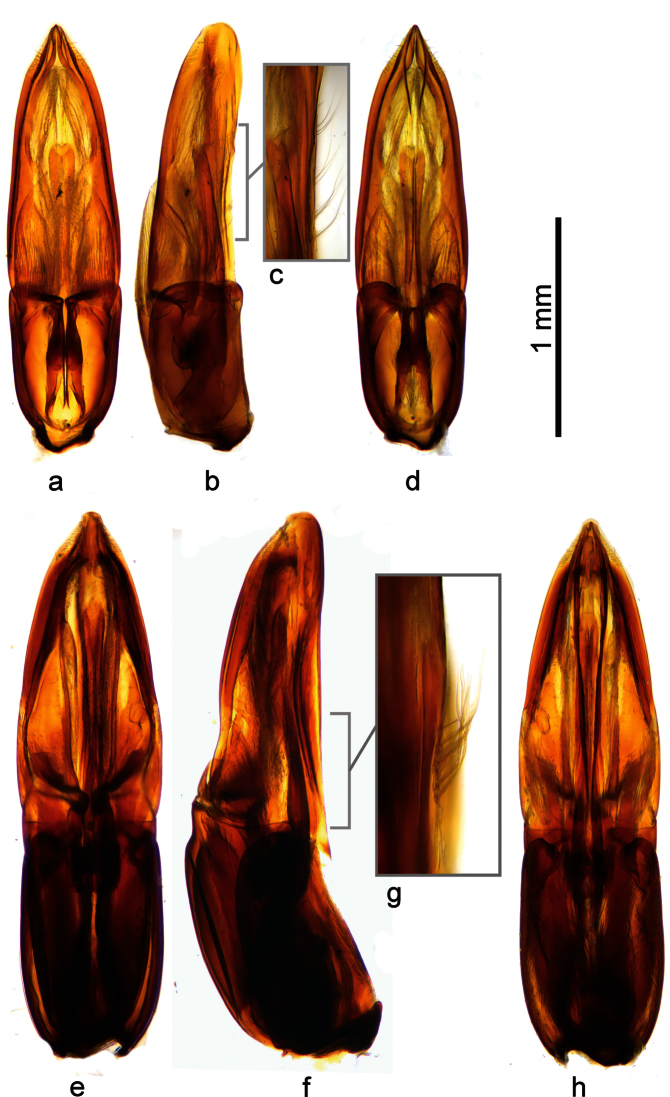
Comparison of the aedeagus of *Berosus truncatipennis* Castelnau, 1840 (**a–d**) and *Berosus quadridens* Chevrolat, 1863 (**e–h**). **a,e** dorsal view **b, f** lateral view **c, g** detail of setae of parameres **d, h** ventral view. Both aedeagi shown to scale.

##### Diagnosis.

Large elongate species, body length 6.2–6.7 mm. Head testaceous with darker central part of clypeus and frons; pronotum pale, with a pair of vaguely defined narrow black longitudinal spots mesally, pronotal punctation darkened; elytra pale with dark elytral striae, interval punctation and variable number of larger dark spots on elytral intervals. Elytral apices each with a large subapical tooth, sutural angle sexually dimorphic, rounded in males, sharply pointed in females. Mesoventral process highly laminar, subtriangular in shape, anterior tooth weakly developed. Abdominal ventrite 1 with median keel developed only between metacoxae. Emargination of abdominal ventrite 5 deeply and narrowly excised in males, shallowly semicircular in females. Aedeagus large, with joint parameres pointed apically, with subbasal tuft of setae on dorsal surface, ventral membranous lobes minute, median lobe slender and long.

##### Differential diagnosis.

*Berosus quadridens* is easily distinguishable from *Berosus truncatipennis* by the relatively larger and more sclerotized aedeagus having stouter and relatively longer phallobase, by ventral face of parameres bearing subbasal tuft of setae (Fig. 8g) (whereas bearing a series of setae (Fig. 8c) in *Berosus truncatipennis*), by relatively longer and narrower median lobe and minute membranous dorsal projections of the parameres (Figs 8e, f, h) (in contrast to moderately large ones present (Figs 8a, b, d) in *Berosus truncatipennis*). The aedeagus of *Berosus quadridens* may resemble that of *Berosus megaphallus* by its large size and presence of subbasal tuft of setae on ventral face of the paramere, but both species distinctly differ by the size and proportions of the phallobase (ca. half as long as the whole aedeagus and very robust in *Berosus megaphallus*; ca. third as long as the whole aedeadus and less robust in *Berosus quadridens*) and by the proportions of the ventral membranous lobe of the paramere (minute in *Berosus quadridens*, nearly as long as paramere in *Berosus megaphallus*). In general, the aedeagus of *Berosus quadridens* looks like an enlarged aedeagus of *Berosus truncatipennis* on the first view, whereas that of *Berosus megaphallus* clearly differs from both *Berosus truncatipennis* and *Berosus quadridens* by the general proportions of its parts. We failed to find any realiable external differences between *Berosus truncatipennis* and *Berosus quadridens*; Van Tassell (1966) indicates the differences in the shape of the apical portion of elytra – these were found rather constant in shape in examined specimens of *Berosus quadridens*, but seem to be very variable in examined specimens of *Berosus truncatipennis* and the character seems to be therefore unrealiable for distinguishing both species at the moment. Based on the differences mentioned above, we confirm that *Berosus quadridens* is a valid species, distinct from *Berosus truncatipennis*.

##### Redescription.

Habitus as in Figs 7a, b. Body length 6.2–6.7 mm. Head, labrum and antennae testaceous. Maxillary palpi testaceous with palpomere 4 dark at apex. Pronotum testaceous with two metallic black central spots developed throughout pronotum. Scutellum testaceous. Elytra testaceous with oblique series of dark brown spots in posterior half and laterally, interval punctuation and elytra striae darkened. Pro- and mesofemora testaceous; metafemora with pubescent portion brown, glabrous portion testaceous.

Head and pronotum with punctures moderately fine and rounded. Elytral striae narrow well impressed. Interstriae fine and flat, bearing spine-like setae on posterior half of elytra. Scutellum with few impressed punctures. Elytral apices bidentate, each bearing a projection on sutural angle and subapically; shape sexually dimorphic, with sutural angle rounded in males (Fig. 7d), sharply pointed in females (Figs 7c). Mesoventral process highly laminar, triangular in shape, anterior tooth barely visible, followed by a convex and smooth ridge (Fig. 7e). Metaventral process raised, triangular in shape, with elongate and deep glabrous median depression; posterolateral angles produced into triangular laminae, posterior projection pointed. Abdominal ventrite 1 with median carina only between metacoxae and with large, deep, rounded lateral depressions. Abdominal ventrite 5 with a deep rounded emargination without tooth in males (Fig. 7g), in females with semicircular apical notch (Fig. 7f). Basal pubescence of meso- and metafemora covering basal two thirds of femoral length, borderline between pubescent and glabrous portion sinuate on meso- femur, straight on metafemur. Protarsus of male with adhesive soles on the two basal tarsomeres, protarsomeres 1-2 thickened, tarsomere 1 longer than tarsomere 2, tarsomere 3 elongate; tarsomere 4 elongate, as long as tarsomeres 1-3 combined. Claws long, slender and curved.

Male genitalia (Figs 8e–h): Phallobase robust, ca. 0.4× as long as whole aedeagus, slightly widening basad in lateral view. Parameres joint mesally, together forming a dish-like structure surrounding median lobe; apical portion rounded in lateral view, pointed in ventral view; ventral portion of each paramere with minute membranous lobe; dorsal face of each paramere with a tuft of setae situated subbasally. Median lobe stick-shaped, reaching to apical 0.75 of paremeres.

##### Taxonomic comments.

Described from Cuba, *Berosus quadridens* was considered endemic to the island, whereas the continental form was supposed to represent the widely distributed South American species *Berosus truncatipennis* (e.g., Zaitzev 1908, Knisch 1924). Based on two females from Cuba (one of which we reexamined in this study), Mouchamps (1963) synonymized *Berosus quadridens* with *Berosus truncatipennis*. This was questioned by Van Tassell (1966) who followed the unpublished opinion of J. Balfour-Browne and considered *Berosus quadridens* as a species separate from *Berosus truncatipennis* occurring not only in Cuba, but also in Central America. The thesis by Van Tassell (1966), and therefore the revalidation of *Berosus quadridens*,remained unpublished and was only adopted without any explanatory comments in the catalogue of Cuban beetles by Peck (2005). Hansen (1999) considered *Berosus quadridens* as a dubious synonym of *Berosus truncatipennis* pending revision (Hansen 1999). Oliva (1989) considered the size and proportions of the genitalia of *Berosus truncatipennis* as geographically variable, being larger and wider in subtropical areas. Recently, Oliva and Short (2012) described the specimens with the large aedeagus from Venezuela and Guyana as a separate species *Berosus megaphallus* Oliva & Short, 2012, but the identity of the Central American and Caribbean specimens remained unsolved.

We were not able to examine the unique type of *Berosus quadridens* from “Cuba”, as it was not found in MNHN after our loan request in 2012. A single species of Cuban *Berosus* matching the original description by Chevrolat (1863) was found in Cuba in our survey; no closely related or similar species was recorded from Cuba. We therefore do not have doubts that the Cuban specimens examined correspond to Chevrolat’s (1863) understanding of *Berosus quadridens*. Moreover, Van Tassell (1966) mentioned that J. Balfour-Browne has examined the type of *Berosus quadridens* and found it to be conspecific with Central American specimens identified previously as *Berosus truncatipennis*. This corresponds with our findings, as we found that all examined Central American specimens of “*Berosus truncatipennis*”are conspecific with the Cuban ones, and clearly differ from the South American species (see Diagnosis above for diagnostic characters).

By confirming the separate species status of *Berosus quadridens*, the originally widely understood *Berosus truncatipennis* is shown to consist of three species: the widely distributed South American *Berosus truncatipennis*, *Berosus quadridens* confined to the Caribbean and Central America, and *Berosus megaphallus* known so far from Venezuela and Guyana. In the material from IRSNB we examined for this study, we have found few specimens from Bolivia (Río Beni) and southern Peru (Ica) which male genitalia are extremely similar to those of *Berosus quadridens* by their large size, strong sclerotization and relatively longer phallobase; however, they seem to differ from *Berosus quadridens* by the presence of the series of setae on the paramere (as in *Berosus truncatipennis*) and the dorsal membranous lobe of the paramere being ca. as long as in *Berosus megaphallus* (examined only in the Bolivian specimen, indistict in dissected Peruan ones). We suppose that these specimens may represent yet another undescribed species of the formerly broadly understood *Berosus truncatipennis*.

##### Habitat.

The Cuban specimens examined in the present work were collected in highly exposed freshwater pools with turbid water, muddy bottom and without cover vegetation. Gundlach (1891) also reports this species from permanent ponds in the Matanzas Province.

##### Distribution.

Based on the specimens examined for this study, we may confirm the occurrence of *Berosus quadridens* for Mexico, Guatemala, Nicaragua, Costa Rica and Cuba. Van Tassell (1966) also maps one record from Panama, but does not cite label data. In Cuba, the species is known from the western (including Isla de la Juventud special municipality) and eastern regions.

#### Berosus trilobus

Chevrolat, 1863

http://species-id.net/wiki/Berosus_trilobus

**Figure 9.**
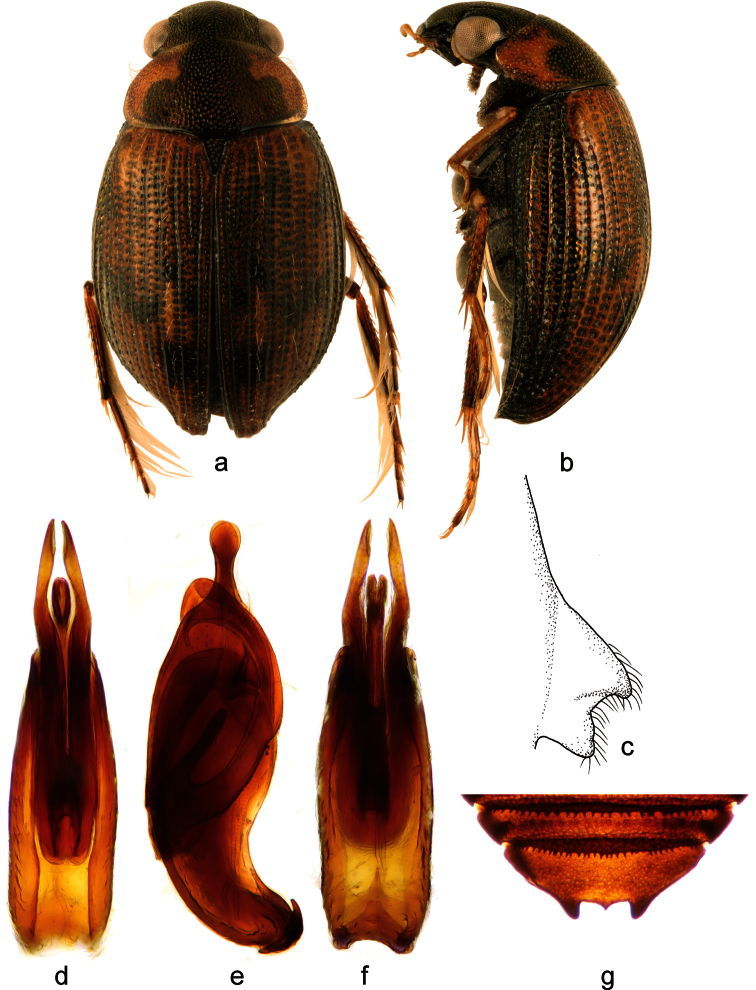
*Berosus trilobus* Chevrolat, 1863. **a** habitus in dorsal view **b** habitus in lateral view **c** mesoventral process in lateral view **d–f** aedeagus (**d** dorsal view **e** lateral view **f** ventral view) **g** abdominal ventrite 5.

Figures 9a–g 11 12a–b

Berosus trilobus Chevrolat, 1863: 207. – Gundlach 1891: 47 (diagnosis and distribution). – Spangler 1973: 354 (distribution). – Spangler 1981: 155 (diagnosis and distribution). – Hansen 1999: 95 (catalogue). – Peck 2005: 48 (checklist). – Epler 2010: 12.24 (notes on distribution).

##### Type locality.

Cuba.

##### Type material examined.

Holotype: female (MNHN): “Berosus / trifidus / Chv. Cuba / … [illegible] // von / G. Hemiosus / Sharp [= of the genus Hemiosus Sharp] // this must be / Chev. unique / type of trilobus / 1966 / PJS [= P. J. Spangler]”.

##### Additional material examined.

**CUBA**: **Sancti Spíritus**:50 exs. (in alcohol) (BSC-E): Topes de Collantes, El Nueve, Río Caburny, 21°55'50"N, 80°00'59"W, 539 m a.s.l., 29.vi.2010, leg. A. Deler-Hernández. **Camagüey**: 19 exs. (in alcohol) (BSC-E): Sierra de Cubitas, Río El Roble, 21°32'53.23"N, 77°46'42.31"W, 55 m a.s.l., 14.iv.2012, leg. A. Deler-Hernández, 00144. **Holguín**: 6 exs. (in alcohol) (BSC-E): Jardín Botánico, Arroyo [stream], 20°51'46.8"N, 76°13'22.8"W, 84 m a.s.l., 07.xii.2008, leg. A. Deler-Hernández, 00074. **Granma**: 7 exs. (in alcohol) (BSC-E): Parque Nacional Turquino, La Platica, 20°00'33.80"N, 76°53'38.47"W, 800 m a.s.l., 29.iii.2012, leg. A. Deler-Hernández, 00143; 12 exs. (dry-mounted) (NMPC): Turquino NP, around La Platica, 20°0.7'N, 76°53.4'W, 880 m a.s.l. [MF24], 25-26.vi.2012 leg. A. Deler-Hernández and M. Fikáček. **Santiago de Cuba**: 6 exs. (in alcohol) (BSC-E): Campo Rico-II, Río Indio, 19°59'54.5"N, 75°32'4.6"W, 150 m a.s.l., 15.ix.2003, leg. A. Deler-Hernández and F. Cala-Riquelme, 00046; 4 exs. (in alcohol) (BSC-E): Gran Piedra, El Olimpo, Arroyo [stream], 20°00'33"N, 75°40'13"W, 820 m a.s.l., 04.viii.2005, leg. A. Deler-Hernández, 00016; 1 ex. (in alcohol) (BSC-E): II Palmas, La Cubana, Laguna temporal-II [temporal pool-II], 20°3'15.48"N, 76°8'3.12"W, 320 m a.s.l., 02.xii.2005, leg. Y. S. Megna, 00086; 30 exs. (in alcohol) (BSC-E): Palma Soriano, Arroyo [stream], 20°06'05"N, 75°58'44"W, 130 m a.s.l., 16.ii.2005, leg. K. Blanco, 00047; 5 exs. (in alcohol) (BSC-E): Guamá, La Mula, Río Turquino, 19°56'57"N, 76°45'36"W, 8 m a.s.l., 21.vi.2005, leg. Y. S. Megna, 00085; 6 exs. (in alcohol) (BSC-E): Guamá, Los Morones, Río Turquino, 19°58'33.6"N, 76°46'4.8"W, 200 m a.s.l., 18.vi.2008, leg. A. Deler-Hernández, 00006; 2 exs. (in alcohol) (BSC-E): San Luis, Dos Caminos, El Vivero, Laguna permanente [permanent pool], 20°11'2.50"N, 75°46'17.7"W, 150 m a.s.l., 01.viii.2008, leg. A. Deler-Hernández, 00028; 3 exs. (in alcohol) (BSC-E): San Luis, Dos Caminos, El Vivero, Río Guaninicú, 20°11'2.50"N, 75°46'17.7"W, 150 m a.s.l., 01.viii.2008, leg. A. Deler-Hernández, 00029; 31 exs. (dry-mounted) (NMPC): El Vivero, 1.6 km E of Dos Caminos, 20°10.8'N, 75°46.4'W, ca. 150 m a.s.l. [MF18], 20–21.vi.2012, leg. A. Deler-Hernández and M. Fikáček; 5 exs. (in alcohol) (BSC-E): Loma del Gato, Chan-Chan, Arroyo [stream], 19°58'27.4"N, 75°53'22.2"W, 353 m a.s.l., 27.vi.2009, leg. A. Deler-Hernández, 00118; 3 exs. (in alcohol) (BSC-E): La Redonda, Río Sevilla, 20°00'54.3"N, 75°45'45.6"W, 15 m a.s.l., 17.v.2009, leg. A. Deler-Hernández, 00154. **Guantánamo**: 36 exs. (in alcohol) (BSC-E) Imías, Yacabo Abajo, Río Yacabo Abajo, 20°06'05"N, 74°69'00"W, 5 m a.s.l., 24.x.2008, leg. A. Deler-Hernández and S. Muñiz, 00091; 20 exs. (in alcohol) (BSC-E): San Antonio del Sur, Macambo, Río Macambo, 20°03'26.9"N, 74°44'15.82"W, 4 m a.s.l., 25.x.2008, leg. A. Deler-Hernández and S. Muñiz, 00055; 16 exs. (in alcohol) (BSC-E): Baracoa-Maisí, Río Yumurí, 20°17'47.76"N, 74°17'39.5"W, 5 m a.s.l., 27.i.2010, leg. A. Deler-Hernández and R. Correa, 00152; 96 exs. (in alcohol) (BSC-E): Baracoa, Yunque, Río Duaba, 20°19'54.40"N, 74°34'9.08"W, 70 m a.s.l., 31.i.2010, leg. A. Deler-Hernández, 00171; 27 exs. (dry-mounted) (NMPC): El Yunque, 2.5-3.3 km SW of campismo popular, 20°19.4'N, 74°34.2'W, ca. 80-100 m a.s.l., 10.vi.2012 [MF02], leg. A. Deler-Hernández and M. Fikáček; 61 exs. (dry-mounted) (NMPC, KSEM): El Yunque, "La Cascada”, ca. 2.1 km SW of campismo, 20°19.9'N, 74°34'W, ca. 60 m a.s.l. [MF07], 12-13.vi.2012, leg. F. Cala-Riquelme, A. Deler-Hernández and M. Fikáček; 2 exs. (dry-mounted) (NMPC): El Yunque, 3.2 km SW of campismo, right tributary of Duaba river, 20°19'N, 74°34'W, ca. 150 m a.s.l. [MF09], 13.vi.2012; leg. A. Deler-Hernández and M. Fikáček; 14 exs. (dry-mounted) (NMPC): El Yunque, in/around campismo popular, 20°20.4'N, 74°32.9'W, ca. 40 m a.s.l. [MF05], 10-13.vi.2012, leg. M. Fikáček; 20 exs. (dry-mounted) (NMPC): PN Alejandro de Humboldt, ca. 1.7 km NW of Santa María, 20°32'N, 74°43'W, ca. 50 m a.s.l. [MF13], 16.vi.2012, leg. A. Deler-Hernández and M. Fikáček; 18 exs. (in alcohol) (BSC-E): Baracoa, Jamal, 20°17'13.9"N, 74°25'33.6"W, 40 m a.s.l., 09.ii.2010, leg. R. Correa, 00169; 1 ex. (in alcohol) (BSC-E): Baracoa, Cabacú, Laguna permanente [permanent pool], 20°19'14"N, 74°28'58"W, 10 m a.s.l., 04.iii.2010, leg. R. Correa, 00170; 6 exs. (in alcohol) (BSC-E) 6 exs. (dry-mounted) (NMPC): Baracoa, Cabacú, Laguna permanente [permanent pool], 20°19'14"N, 74°28'58"W, 10 m a.s.l., 16.iii.2010, leg. R. Correa , 00164; 1 ex. (in alcohol) (BSC-E): La Marsella, Río Guaso, 20°26'22"N, 74°42'31"W, 60 m a.s.l., 26.i.2004, leg. Y. S. Megna , 00173; 3 exs. (in alcohol) (BSC-E): Baracoa, Loma de los Guineos, Arroyo [stream], 20°19'38.38"N, 74°35'35.37"W, 530 m a.s.l., 07.iv.2012; leg. A. Deler-Hernández, 00177. **Without precise locality**:2 exs. (dry-mounted) (NMPC): “O. Koechin / Cuba // Collectio / Dr. Jureček / H. Jurečková”; 1 ex. (dry-mounted) (MNHN): “1542 / 1798”. **DOMINICAN REPUBLIC:** 25 exs. (dry-mounted) (KSEM, NMPC): near Hato Mayor, creek off Ruta 103, 02.xi.2000, leg. A. E. Z. Short.

**Published Cuban records:** **Cuba:** without specified locality(Gundlach, 1891). **Pinar del Río**: Quemado de Pineda (Spangler 1981). **Sancti Spíritus**: Río Caburny near Topes de Collantes (Spangler 1973); Arroyo Vegas Grande near Topes de Collantes (Spangler 1973). **Camagüey**: Río El Manantiales (Spangler 1981). **Holguín**: Arroyo Jarahueca (Spangler 1981). **Santiago de Cuba**: II Frente, Sabanilla, Arroyo La Poa (Spangler 1981); II Frente, Arroyo Jarahueca (Spangler 1981); Contramaestre, Pozo Caliente, Río Contramaestre (Spangler 1981); II Frente, Sabanilla, Río Mayarí (Spangler 1981); II Frente, Río Ceiba affl. Río Mayarí (Spangler 1981); III Frente, Río Brazo Seco (Spangler 1981); III Frente, Matías, Río Mogote (Spangler 1981). **Guantánamo**: Maisí, La Tinta, Río Baracoa (Spangler 1973, 1981); Niceto Pérez, Sierra de Canasta, Arroyo de los Berros (Spangler 1981); Río Miel at Baracoa (Spangler 1973); Baracoa, Yumurí, Río Yumurí (Spangler 1981).

##### Diagnosis.

Small widely elongate species, body length 3.2–3.7 mm. Head dark, metallic; pronotum pale laterally, with large trilobite central dark spot, pronotal punctation not darkened laterally; elytra pale with dark intervals 8-10 and large transverse dark spots on posterior half of elyttral intervals 1-7. Elytral apices without subapical tooth, bumpy along suture subapically. Mesoventral process highly laminar, rectangular with large anterior and posterior teeth. Abdominal ventrite 1 with median keel throughout its length. Emargination of abdominal ventrite 5 rectangular with a median tooth. Median lobe of the aedeagus with long basal projection and beak-like apex in lateral view.

**Differential diagnosis.** For diagnostic characters and difference from *Berosus chevrolati*, see the latter species.

##### Redescription.

Habitus as in Figs 9a, b. Body length 3.2–3.7 mm. Body short and wide, moderately convex in lateral view. Labrum black, dorsum of head melanic with strong metallic luster. Antennae testaceous. Maxillary palpi testaceous with palpomere 4 dark at apex. Pronotum testaceous with unpaired metallic black spot, the spot expanding laterad posteriorly, and hence trilobite in general shape. Elytra testaceous with small ill-defined dark brown spots on disc and, a broad metallic dark area throughout lateral portion. Pro-, meso- and metafemora with pubescent portion dark brown, glabrous portion testaceous.

Head with moderately large and rounded punctures. Pronotum with punctures slightly larger than on head. Scutellum with few impressed punctures. Elytral striae distinctly impressed; intervals flat and wide, irregular long setae on elytra; spine-like setae absent. Elytral apices entire and rounded in both sexes; highly bumpy along suture, depressed laterally on sides. Mesoventral process raised, rectangular in shape, with hood-like anterior tooth, posterior tooth large (Fig. 9c). Metaventral process widely rectangular, with large and deep elongate glabrous median depression; posterolateral portions bulge-like, posterior projection pointed. Abdominal ventrite 1 with median carina throughout the length. Abdominal ventrite 5 with rectangular emargination bearing broad and sharp median tooth (Fig. 9g). Meso- and metafemora with pubescence covering basal 0.7 of total length, borderline between pubescent and glabrous portion sinuate. Protarsus of male with adhesive soles on tarsomeres 1-2, tarsomeres 1-2 distinctly thickened, tarsomere 3 elongate; tarsomere 4 2× as long as tarsomere 3. Claws long, slender, slightly arched.

Male genitalia (Figs 9d-f). Phallobase ca. 0.7× total length of aedeagus. Parameres in lateral view wide basaly, narrowing subapically and apically projecting into rounded apex, lacking setae. Median lobe G-shaped in lateral view, with long basal projection directing apicad; apex wide, beak-shaped in lateral view.

##### Distribution.

Dominican Republic and Cuba. The species was until now considered as Cuban endemic (e.g., Hansen 1999, Peck 2005), although Van Tassell (1966) mentioned specimens from the Dominican Republic. We are here confirming the occurrence of the species in the Dominican Republic based on recently collected specimens deposited in KSEM.

##### Habitat.

In our survey, the specimens of *Berosus trilobus* were collected usually in streams and rivers with stony or sandy bottom, clear water and with or without aquatic vegetation (Figs 11a, b), although once it has also been collected in a temporary pool with stony-muddy bottom, abundant organic matter, turbid water and rich submerged vegetation. *Berosus trilobus* is found in elevations ranging from sea level to ca. 850 m a.s.l.

#### Berosus undatus

(Fabricius, 1792)

http://species-id.net/wiki/Berosus_undatus

Figures 10a–i 11

Hydrophilus undatus Fabricius, 1792: 185.
Berosus undatus (Fabricius, 1792). Synonymy: Gemminger and Harold 1868: 485. – Van Tassell 1966: 74 (unpublished PhD thesis: redescription, identification key). – Spangler 1981: 156 (diagnosis and distribution). – Hansen 1999: 82 (catalogue). – Peck 2005: 48 (checklist). – Epler 2010: 12.24 (notes on distribution). – Deler-Hernández and Cala-Riquelme 2010: 73 (diagnosis, distribution, identification key). For complete synonymy and references see Hansen (1999).

##### Type locality.

“America meridionalis”.

**Material examined. CUBA**: **Las Tunas**: 1 ex. (in alcohol) (BSC-E): Las Cuarenta, 20°00'9.72"N, 76°57'48.6"W, 100 m a.s.l., 27.xi.2004, leg. Y. S. Megna, 00045. **Granma**: 2 exs. (in alcohol) (BSC-E): Cauto Cristo, Laguna permanente-II [permanent pool-II], 20°33'33.1"N, 76°28'44"W, 44 m a.s.l., 06.iii.2004, leg. L. Chávez, 00053. **Santiago de Cuba**: 4 exs. (in alcohol) (BSC-E): Laguna Juraguá, 19°56'30.8"N, 75°40'21.3"W, 22 m a.s.l, 17.ix.2003, leg. Y. S. Megna, 00044; 1 ex. (in alcohol) (BSC-E); 3 exs. (dry-mounted) (NMPC): Palma Soriano, Monte Barranca, 20°20'13.5"N, 76°1'11.6"W, 203 m a.s.l., 05.xii.2007, leg. A. Deler-Hernández, 00054; 1 ex. (dry-mounted) (NMPC): Palma Soriano 20°06'05"N, 75°58'44"W, 130 m a.s.l. 01.v.2005, leg. K. Blanco; 2 exs. (in alcohol) (BSC-E): La Maya, Los Reinaldos, Laguna temporal [temporal pool], 20°11'12"N, 75°31'43"W, 100 m a.s.l., 17.iii.2006, leg. Y. S. Megna, 00088. **Guantánamo:** 1 ex. (in alcohol) (BSC-E): Imías,Yacabo Abajo, Laguna temporal [temporal pool], 20°03'33.1"N, 74°42'29.9"W, 6 m a.s.l., 24.x.2008, leg. A. Deler-Hernández and S. Muñiz, 00060; 3 exs. (in alcohol) (BSC-E): San Antonio del Sur, Macambo, río Macambo, remanso [backwater], 20°03'26.9"N, 74°44'15.82"W, 4 m a.s.l., 25.x.2008, leg. A. Deler-Hernández and S. Muñiz, 00059.

**Published Cuban records:** **Santiago de Cuba**: Laguna Juraguá (Spangler 1981); Siboney (Spangler 1981). **Holguín**: Gibara, La Aguada (Spangler 1981).

**Figure 10.**
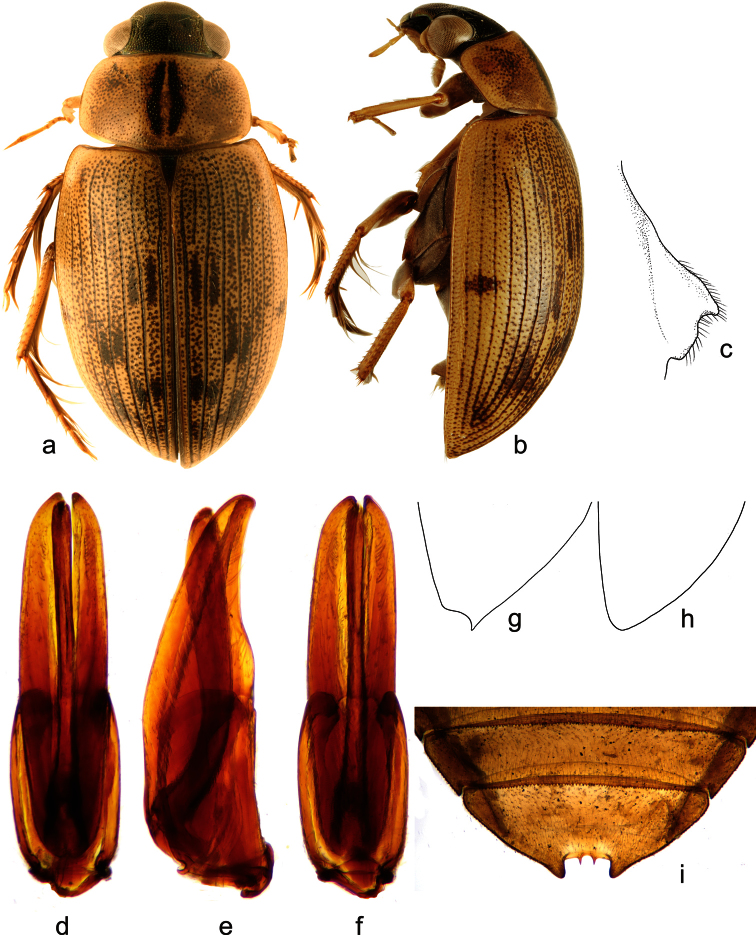
*Berosus undatus* (Fabricius, 1792). **a** habitus in dorsal view **b** habitus in lateral view **c** mesoventral process in lateral view **d–f** aedeagus (**d** dorsal view **e** lateral view **f** ventral view) **g** apex of male elytron **h** apex of female elytron **i** abdominal ventrite 5.

**Figure 11.**
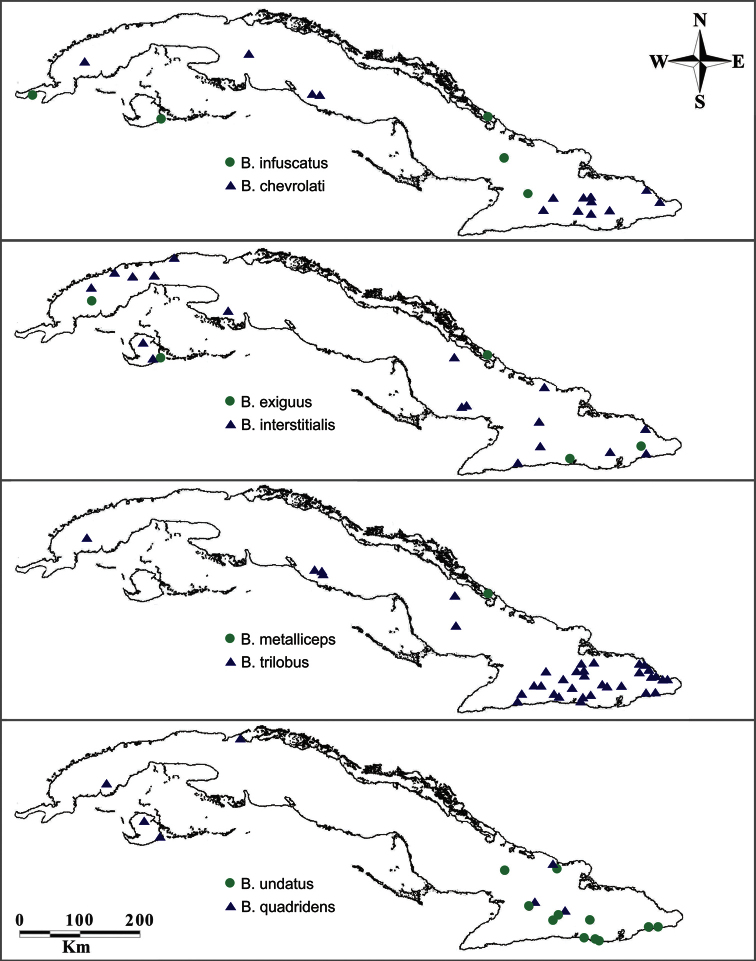
Known distribution of *Berosus* species in Cuba (includes our as well as historical records).

**Figure 12.**
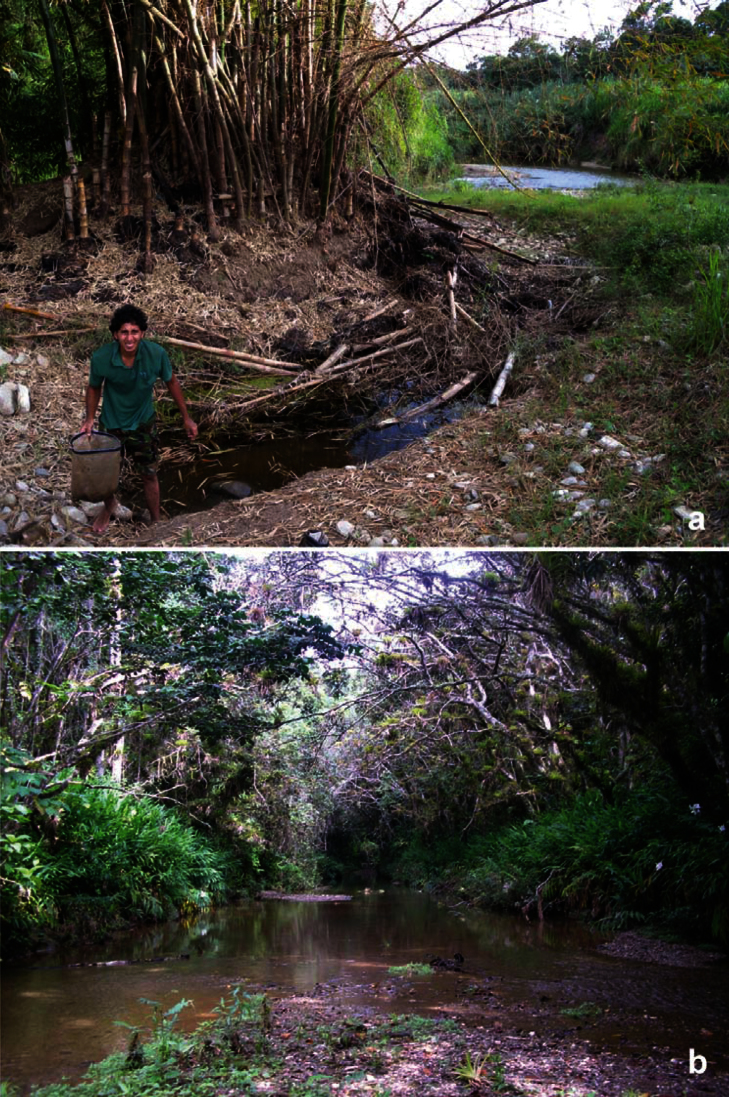
Localities of Cuban *Berosus* **a** Deler-Hernández collecting *Berosus chevrolati* and *Berosus trilobus* in a deep pool on side of a lowland river in Dos Caminos (June 2012) **b** locality of *Berosus trilobus* near Topes de Collantes (June 2010).

##### Diagnosis.

Habitus as in Figs 10a, b. Body length 6.3–7.2 mm. Head metallic black; pronotum pale with a pair of closely arranged elongate longitudinal black spots mesally, pronotal punctuation darkened; elytra pale with darkened striae and interval punctuation, plus with larger elongate dark spots on posterior half of intervals 1-4 and at midlength of intervals 8-10. Elytral apices with subapical spine in male (Fig. 10g), entire and rounded in female (Fig. 10h). Mesoventral process lowly laminar with large tooth directed posteriad (Fig. 10c). Abdominal ventrite 1 with median keel developed only between metacoxae. Emargination of abdominal ventrite 5 rectangular, with two broad and short medial teeth (Fig. 10i). Aedeagus (Figs 10d-f) with median lobe ca. as long as parameres, lateral margins of parameres subparallel except apically; median lobe narrow in dorsal view, slightly wider in lateral view.

##### Distribution.

USA (Texas), Mexico, Lesser Antilles (Hansen, 1999) and Cuba.

##### Habitat.

Cuban specimens were collected in permanent and temporary pools as well as from running waters with clear or turbid water, having or lacking aquatic vegetation.

### Key to identification of Cuban *Berosus*

**Table:** 

1	First abdominal ventrite carinate along its entire length or large part of it. Small to medium sized species (3.0-4.6 mm). Pronotum testaceous, with unpaired median black spot (Figs 1a, 9a) or without any dark spots (Fig. 2a)	2
–	First abdominal ventrite carinate only basally. Medium sized to large species (4.1-7.2 mm). Pronotum pale or testaceous with a pair of median black spots (Figs 3a, 4a, 6a, 7a, 10a), or pale without dark spots (Fig. 5a)	4
2	Head black. Pronotum and elytra with dark spots. Dorsal surface coarsely punctate. Mesoventral process subquadrate. First abdominal ventrite with median carina throughout the length. Median lobe of the aedeagus without subapical dorsal series of setae	3
–	Head testaceous; pronotum and elytra without dark spots (Fig. 2a). Meso-ventral process triangular (Figs 2b-c). First abdominal ventrite with median carina reaching the midlength. Aedeagus with median lobe bearing two series of long setae setae subapically on dorsal surface (Fig. 2e)	*Berosus exiguus* (Say)
3	Dark pronotal spot large and trilobate, narrow anteriorly and very wide posteriorly (Fig. 9a). Subapical area of each elytron forming a bump (Figs 9a-b). Apex of the median lobe beak-shaped in lateral view, basal projection of the median lobe long (Fig. 9e)	*Berosus trilobus* Chevrolat
–	Dark pronotal spot narrow, situated mesally, not widened posteriad (Fig. 1a). Subapical area of each elytron without a distinct bump (Figs 1a-b). Apex of the median lobe rounded in lateral view, basal projection of the median lobe short (Fig. 1e).	*Berosus chevrolati* Zaitzev
4	Head metallic black to black. First abdominal ventrite without lateral depressions, emargination of abdominal ventrite 5 rectangular, without distinct sexual dismorphism. Parameres separated from each other. Elytral apex with or without subapical spine	5
–	Head testaceous (except mesally in some cases, Figs 7a–b). First abdominal ventrite with lateral depressions; emargination of abdominal ventrite 5 deeply or shallowly circular, sexually dimorphic (Figs 7f–g). Parameres joined mesally into a common dish-like structure (Figs 8e–h). Elytral apex with subapical spine in both sexes (Figs 7c–d)	*Berosus quadridens* Chevrolat
5	Pronotal disc without spots (Fig. 5a) or with small submesal anterior spots (Fig. 6a), never with a pair of mesal elongate large dark spots throughout the pronotal length. Apical emargination of abdominal ventrite 5 without tooth (Fig. 5g) or with a single medial tooth (Fig. 6g). Median lobe longer that parameres	6
–	Pronotal disc with a pair of narrow elongate metallic central black spots. Apical emargination of abdominal ventrite 5 with two medial teeth. Median lobe shorter that parameres	7
6	Elytral striae distinctly darkened, elytral disc without numerous darker spots (Figs 5a–b). Apical emargination of abdominal ventrite 5 without median tooth (Fig. 5g). Median lobe of the aedeagus very long, spatulate apically in dorsal view, sinuate in lateral view (Figs 5d–f)	*Berosus metalliceps* Sharp
–	Elytral series not darkened, each elytron with several darker spots on the disc (Figs 6a-b). Apical emargination of abdominal ventrite 5 with a broad and short median tooth (Fig. 6g). Median lobe of the aedeagus slender and apex, pointed in dorsal view, slightly arcuate in lateral view (Figs 6d–f)	*Berosus peregrinus* (Herbst)
7	Body size less than 6.0 mm. Elytral apices entire (without subapical spines) in both sexes. Phallobase longer than a half of total length of the aedeagus	8
–	Body size more than 6.3 mm. Elytral apices sexually dimorphic, with subapical spine in males (Fig. 10g) and rounded in females (Fig. 10h). Phallobase shorter than a half of the total length of the aedeagus (Figs 10d–f)	*Berosus undatus* (Fabricius)
8	Pronotum without mesh-like microsculpture on interstices. Mesoventral process with very small tooth (Fig. 4c). Posterolateral angles of metaventral process triangular. Aedeagus narrow, lateral margins of parameres subparallel, base of each paramere with a conspicuous tooth (Figs 4d–f)	*Berosus interstitialis*
–	Pronotum with strong mesh-like microsculpture on interstices. Mesoventral process with larger tooth (Fig. 3c). Posterolateral angles of metaventral process with rounded laminae. Parameres sinuate along lateral margins, base of parameres without conspicuous teeth (Figs 3d–f)	*Berosus infuscatus*

## Supplementary Material

XML Treatment for Berosus

XML Treatment for Berosus chevrolati

XML Treatment for Berosus exiguus

XML Treatment for Berosus infuscatus

XML Treatment for Berosus interstitialis

XML Treatment for Berosus metalliceps

XML Treatment for Berosus peregrinus

XML Treatment for Berosus quadridens

XML Treatment for Berosus trilobus

XML Treatment for Berosus undatus
